# Other PHAC publications

**DOI:** 10.24095/hpcdp.44.11/12.05

**Published:** 2024-11

**Researchers from the Public Health Agency of Canada also contribute to work published in other journals and books. Look for the following articles published in 2024:**

Aflaki K, Ray JG, Edwards W, Scott H, Arbour L, Darling EK, […] **Dzakpasu S**. Maternal deaths by suicide and drug overdose in two Canadian provinces; retrospective review. J Obstet Gynaecol Can. 2024;46(8):102581. https://doi.org/10.1016/j.jogc.2024.102581

Agung Y, Hladkowicz E, **Boland L**, Moloo H, Lavalle LT, Lalu MM, et al. Frailty and decisional regret after elective noncardiac surgery: a multicentre prospective cohort study. Br J Anaesth. 2024 [in press]. https://doi.org/10.1016/j.bja.2024.08.001

**Borhani P**, Walker KL, **Butler GP, Lavergne V, Contreras G, Prince SA**. Measuring active transportation on national health surveys in Canada from 1994 to 2020. J Phys Act Health. 2024;21(8):817-28. https://doi.org/10.1123/jpah.2023-0429

Brodkin S, **Sibalis A**, Bedard AV, Deluca A, Suri A, Balter A, et al. Mental health needs assessment for youth in out of school programs: a scoping review. J Community Appl Soc Psychol. 2024;34(5):e2873. https://doi.org/10.1002/casp.2873

**Dam D, Chen M, Rees EE, Cheng B, Sukkarieh L, McGill E, Tehami Y, Bellos A, Edwin J, Patterson K**. Risk factors associated with the intensity of COVID-19 outbreaks in Canadian community settings: a retrospective analysis of outbreak-level surveillance data. BMC Public Health. 2024;24(1):2409. https://doi.org/10.1186/s12889-024-19853-4

Dawe R, Penashue J, Knight JC, Pike A, Benuen MP, Qupee A, **Pollock NJ**. Mortality in Innu communities in Labrador, 1993–2018: a cross-sectional study of causes and location of death. Int J Circumpolar Health. 2024;83(1):2378581. https://doi.org/10.1080/22423982 .2024.2378581

Denis-Robichaud J, **Rees EE**, Daley P, Zarowsky C, **Diouf A**, Nasri BR, et al. Linking opinions shared on social media about COVID-19 public health measures to adherence: repeated cross-sectional surveys of Twitter use in Canada. J Med Internet Res. 2024;26:e51325. https://doi.org/10.2196/51325

**Drysdale M**, Skinner K, **Lazarescu C, Couture A, Young S, Idzerda L**. Initiatives and exposures associated with food security in remote and isolated communities: a scoping review. Rural Remote Health. 2024;24(3):8627. https://doi.org/10.22605/rrh8627

**Dzakpasu S**, Li Z, Ayoub A, Wei SQ, Auger N. Validation of database autopsy for review of pregnancy-associated deaths in Canada. J Obstet Gynaecol Can. 2024;46(9):102611. https://doi.org/10.1016/j.jogc.2024.102611

**Dzakpasu S, Nelson C**, Darling EK, Edwards W, Murphy PA, Scott H, et al. Trends in rate of hypertensive disorders of pregnancy and associated morbidities in Canada: a population-based study (2012–2021). CMAJ. 2024;196(26):E897-904. https://doi.org/10.1503/cmaj.231547

**Enns A**, Abele B, Bowes M, Murray R, **Rotondo J, VanSteelandt A**. Underlying polysubstance classes and associated sociodemographic characteristics and health histories among people who died from substance-related acute toxicity in Canada: a latent class analysis. Int J Ment Health Addict. 2024. https://doi.org/10.1007/s11469-024-01378-x

**Ferguson AL,** Erwin E, Sleeth J, Symonds N, Chard S, Yuma S, et al. An implementation evaluation of the Smartphone-Enhanced Visual Inspection with Acetic acid (SEVIA) program for cervical cancer prevention in urban and rural Tanzania. Int J Environ Res Public Health. 2024;21(7):878. https://doi.org/10.3390/ijerph21070878

Gabet S, Levasseur A, Thierry B, **Wasfi R**, Kestens Y, Moullec G, et al. Household and housing determinants of sleep duration during the COVID-19 pandemic: results from the COHESION Study. Sleep Health. 2024;10(5):602-9. https://doi.org/10.1016/j.sleh.2024.05.008

Larouche R, Abadi MRH, Aubert S, Bhawra J, Brazo-Sayavera J, Carson V, […] **Roberts K**, et al. Development and validation of the Global Adolescent and Child Physical Activity Questionnaire (GAC-PAQ) in 14 countries: study protocol. BMJ Open. 2024;14(7):e082275. https://doi.org/10.1136/bmjopen-2023-082275

Lessard , **O’Brien N**, Panaite A, Leclaire M, Castonguay G, Rouly G, et al. Can you be a peer if you don’t share the same health or social conditions? A qualitative study on peer integration in a primary care setting. BMC Primary Care. 2024;25(1):298. https://doi.org/10.1186/s12875-024-02548-5

**MacKay H, Gretton JD, Chyderiotis S, Elliott S, Howarth A, Guo C, Mastroianni A, […] Morrissey MD**. Confidence and barriers: analysis of factors associated with timely routine childhood vaccination in Canada during the COVID-19 pandemic. Vaccine. 2024; 42(24):126236. https://doi.org/10.1016/j.vaccine.2024.126236

Mbutiwi FIN, Yapo APJ, Toirambe SE, **Rees E, Plouffe R**, Carabin H. Sensitivity and specificity of International Classification of Diseases algorithms (ICD-9 and ICD-10) used to identify opioid-related overdose cases: s systematic review and an example of estimation using Bayesian latent class models in the absence of gold standards. Can J Public Health. 2024. https://doi.org/10.17269/s41997-024-00915-4

**Nelson CRM**, Ray JG, Auger N, Moore AM, Little J, Murphy PA, […]; **Canadian Perinatal Surveillance System, Public Health Agency of Canada**. Neonatal adverse outcomes among hospital livebirths in Canada: a national retrospective study. Neonatology. 2024:1-8. https://doi.org/10.1159/000540559

**Nunn A**, Perri AM, Gordon H, Harding JPD, Loo CKJ, Tuinema J. Opioid-related deaths in Northern Ontario in the early COVID-19 pandemic period. Can J Public Health. 2024. https://doi.org/10.17269/s41997-024-00906-5

**Stevens A, Hersi M, Garritty C**, Hartling L, Shea BJ, Stewart LA, et al. Rapid review method series: interim guidance for the reporting of rapid reviews. BMJ Evid Based Med. 2024:CD013632. https://doi.org/10.1136/bmjebm-2024-112899

**Zutrauen S, Cheesman J, McFaull SR**. Pediatric injuries and poisonings associated with detergent packets: results from the Canadian Hospitals Injury Reporting and Prevention Program (CHIRPP), 2011–2023. Inj Epidemiol. 2024;11(1):31. https://doi.org/10.1186/s40621-024-00513-5

